# Extending the Impact of Mood Disorder Care Through Treatment Optimization and Accessibility

**DOI:** 10.1177/07067437251374563

**Published:** 2025-09-18

**Authors:** Peter Giacobbe, Muhammad Ishrat Husain

**Affiliations:** 1 71545 Sunnybrook Health Sciences Centre, Toronto, ON, Canada; 2 Department of Psychiatry, Temerty Faculty of Medicine, 7938 University of Toronto, Toronto, ON, Canada; 3 Krembil Brain Institute and Centre for Mental Health, University Health Network, Toronto, ON, Canada; 4 Temerty Centre for Therapeutic Brain Intervention, Campbell Family Mental Health Research Institute, 7978 Centre for Addiction and Mental Health, Toronto, ON, Canada

**Keywords:** major depressive disorder, lithium, barriers to treatment, electroconvulsive therapy, patient engagement

Although there are effective treatments available, evidence suggests the majority of those with mood disorders in many parts of the world including in Canada do not receive minimally adequate guideline concordant care. Moving the needle forward at a public health level will require research focusing on the dual goals of both increasing the number of evidence based, effective and novel treatments available and improving accessibility to them. An effective treatment that is not accessible or acceptable by those that need it, is like the proverbial tree that falls in the forest without anyone around to hear it.

A series of articles in this month's issue seek to improve mood disorder outcomes through research seeking to enhance the therapeutic effects of established evidence-based treatments or minimizing side-effects, as well as by incorporating the patient perspective on factors that are important to them in order to identify and reduce barriers to accessing effective treatments. Working on both approaches can allow these optimized treatments to reach more people who may benefit from them.

Electroconvulsive therapy (ECT) is a prime example of the promise of this strategy. Although ECT is an established treatment for those with severe or refractory forms of psychiatric illness, studies continue to report that it is underutilized. The factors associated with its lower than indicated use are multifactorial and include not only identified gaps in pedagogical methods^1^ but also the enduring concerns expressed by some patients and their families of potential cognitive impairment with ECT. Although there is evidence that selecting certain ECT parameters, such as using ultra-brief pulse right unilateral ECT, can minimize the cognitive side-effects experienced by patients, there is certainly room for further refinements in this area in terms of the technique as well as opportunities to stimulate relevant brain areas with other methods. In this context, magnetic seizure therapy (MST), an alternative form of convulsive therapy that generates a therapeutic seizure under anaesthesia through the delivery of focused magnetic pulses as opposed to direct electrical stimulation of the brain, has demonstrated the ability to preserve the potent antidepressant effects similar to ECT while minimizing any short-term cognitive side-effects.

In the systematic review and meta-analysis performed by Prillo and colleagues regarding the clinical and cognitive effects of MST across a wide-variety of psychiatric disorders, pooled evidence from 17 studies suggests that the antidepressant effects of MST are equivalent to those of ECT.^2^ Furthermore, in the 7 studies which directly compared the cognitive effects of MST to ECT, all 7 studies reported superior outcomes on cognitive domains in those receiving MST compared to ECT. The most robust findings were in the time to re-orientation following treatment, which was consistently shorter in the MST groups. The available data are more limited regarding the efficacy of MST in other psychiatric disorders, with preliminary evidence that psychotic disorders are responsive to MST, suggesting that the clinical profile of MST is more similar to ECT than repetitive transcranial magnetic stimulation (rTMS).

Further replication in large-scale randomized clinical trials (RCTs) and a greater accessibility of the MST machines would certainly allow more people with a severe and/or refractory depressive and/or psychotic episode who are reluctant to receive ECT to access MST, an alternative form of convulsive therapy. Although the data about MST as reviewed in this issue may potentially reflect that it has a more favourable balance of positive clinical and side-effects profile than ECT, it is important to note that most studies report that more treatments are required with MST to achieve its antidepressant effect compared to ECT, often double.

However, for individuals where multiple anaesthetic inductions and time to achieve a response are not rate-limiting steps in their clinical care, MST may represent an important alternative to ECT.

Although it has been reported that high-frequency rTMS to the left DLPFC is not associated with deleterious cognitive side-effects, a meta-analysis of left DLPFC rTMS delivered transdiagnostically to those with a variety of psychiatric or neurological disorders reported no to minimal effects on improving cognitive domains,^3^ in contrast to the medium effect sizes for reducing depressive symptoms. The potential pro-cognitive effects of rTMS would certainly help to provide more options for those experiencing cognitive symptoms of their mood disorder, as this is often not adequately addressed by psychotropic medications; further, cognitive symptoms in patients with mood disorders are often associated with significant impairment in quality of life and functioning. DeMayo and colleagues present data on a pharmacological strategy to enhance rTMS, namely the addition of d-cycloserine, an NMDA agonist which putatively targets the glutamatergic systems associated with long-term potentiation and neuroplasticity.^4^ In their placebo-controlled trial of d-cycloserine added to open-label iTBS rTMS, they found the group that received active d-cycloserine demonstrated enhanced subjective and objective cognitive improvements, especially in working memory. In the group of patients with major depressive disorder (MDD) that received d-cycloserine augmentation, their cognitive performance post-treatment was similar to healthy controls. These findings which augment and extend the reach of left DLPFC rTMS beyond established antidepressant effects to pro-cognitive effects has important clinical implications. Given that the doses of d-cycloserine used to achieve the effects seen in this study are very well-tolerated and the addition of pharmacological adjunct does not alter the amount of the time per session or treatment intensity of the iTBS rTMS, the potential translatability of this finding to clinical practice should be rapid.

A study by Ng and colleagues also extends the reach of rTMS further by providing evidence that more people may benefit from receiving it than previously believed.^5^ This analysis evaluated the effect of comorbid posttraumatic stress disorder (PTSD) on antidepressant outcomes in individuals with MDD both with and without active PTSD symptoms. A history of trauma is a frequent factor in those presenting for rTMS and has been hypothesized as contributing to more refractory forms of MDD. Although a common comorbidity of MDD, individuals with PTSD are not frequently represented in RCTs of rTMS for MDD, leading to a lack of clarity about the impact of PTSD on antidepressant outcomes with this treatment modality. In a real-world sample of patients presenting in a depressive episode of moderate to severe intensity and treated with high-frequency rTMS to the left DLPFC, Ng and colleagues report that a history of trauma with active PTSD symptoms did not reduce the magnitude of the antidepressant effect of rTMS. This important clinical finding suggests that those with PTSD who presents in a depressive episode should not be excluded from receiving this treatment.

Although the study confirmed the antidepressant effects of high-frequency rTMS to the left DLPFC for those with MDD with or without PTSD, the extent to which this protocol directly or indirectly ameliorated PTSD symptoms was not reported. Future studies are required to determine if those with MDD and PTSD could benefit from “enhanced” rTMS protocols, adding other forms of stimulation to high-frequency rTMS to the left DLPFC to improve the core features of PTSD as well.

In addition to optimization of existing evidence-based treatments, exploring factors which influence patient perspectives on treatment options may yield valuable actionable insights to improve outcomes. Giving a voice to those with lived life experience in research studies can help mental health care providers and systems to understand what is important to those receiving treatment, guide the selection of options and decrease barriers to care. Strawbridge and colleagues describe the experiences of patients purchasing over-the counter lithium supplements (LiS), which when compared to conventional forms of lithium that require a prescription, are used at lower doses and do not require regular monitoring of serum levels and other blood indices.^6^ Ostinelli and colleagues present their work on the PETRUSHKA (Personalize antidEpressant Treatment foR Unipolar depreSsion combining individual cHoices, risKs and big datA) tool, an online, evidence-based patient decision aid to support shared clinical decision-making to select an antidepressant medication.^7^ To create the tool, the authors used available patient-level data from 130 RCTs totalling over 40,000 participants and real-world health records of over 25 million primary care patients in England. As the critical next step, patients are then requested to input their personalized ranking of the top “deal-breaker” side effects that they wish to avoid. Based on the information provided, the output the PETRUSHKA tool is an anonymized presentation of the top 3 recommended antidepressant medications to guide the subsequent doctor–patient discussion. The strengths of the tool include it being a web-based application allowing for it to broadly accessible in a wide range of geographic and clinical settings, perhaps most impactfully in primary care. Although promising, the next stage of validation will be studies to demonstrate that the use of the PETRUSHKA tool, with its emphasis on individualized factors that are important to the patient, can decrease the rates of premature discontinuation of antidepressants and improve outcomes. Future iterations of this tool should be able to incorporate large databases of the pharmacogenetic and biological factors associated with responses to specific antidepressant medications, as they become available, to further hone its precision.

Lastly, Chisamore and colleague present data comparing outcomes in adults with treatment resistant depression who are on antidepressant medications and those required to undergo an antidepressant washout ahead of a course of psilocybin assisted psychotherapy (PAP).^8^ This post-hoc analysis seeks to determine whether the discontinuing antidepressant medication attenuates the response to PAP or if this is overshadowed by the possible risk of worsening emotional health due to either an antidepressant discontinuation syndrome or a relapse in the depressive illness. The authors report no differences between these groups in PAP's positive clinical effects on mood or anxiety nor frequency of adverse effects. These data should provide reassurance for patients and clinicians, potentially extending the reach of this emerging treatment for future clinical implementation.

As the articles in the current issue demonstrate, the pursuit of improving outcomes for those with mood disorders is multifaceted and dynamic. From optimizing established treatments like rTMS, to integrating patient preferences in treatment selection through tools like PETRUSHKA, and exploring novel approaches like MST and PAP, the field is actively striving to enhance both treatment effectiveness and accessibility. These advancements offer hope for a future where more individuals receive timely, effective and personalized treatment for mood disorders, leading to improved outcomes.
